# Supplementary material for: Medically Actionable Secondary Findings from Whole-Exome Sequencing (WES) Data in a Sample of 3972 Individuals
Source: Int J Mol Sci. 2025 Apr 9;26(8):3509. doi: 10.3390/ijms26083509 (PMC12027037; doi:10.3390/ijms26083509)
Supplement: Supplementary file 1 [file ijms-26-03509-s001.zip › Figure S1. Pipeline used for processing and generating multi-sample VCF file_.pdf]

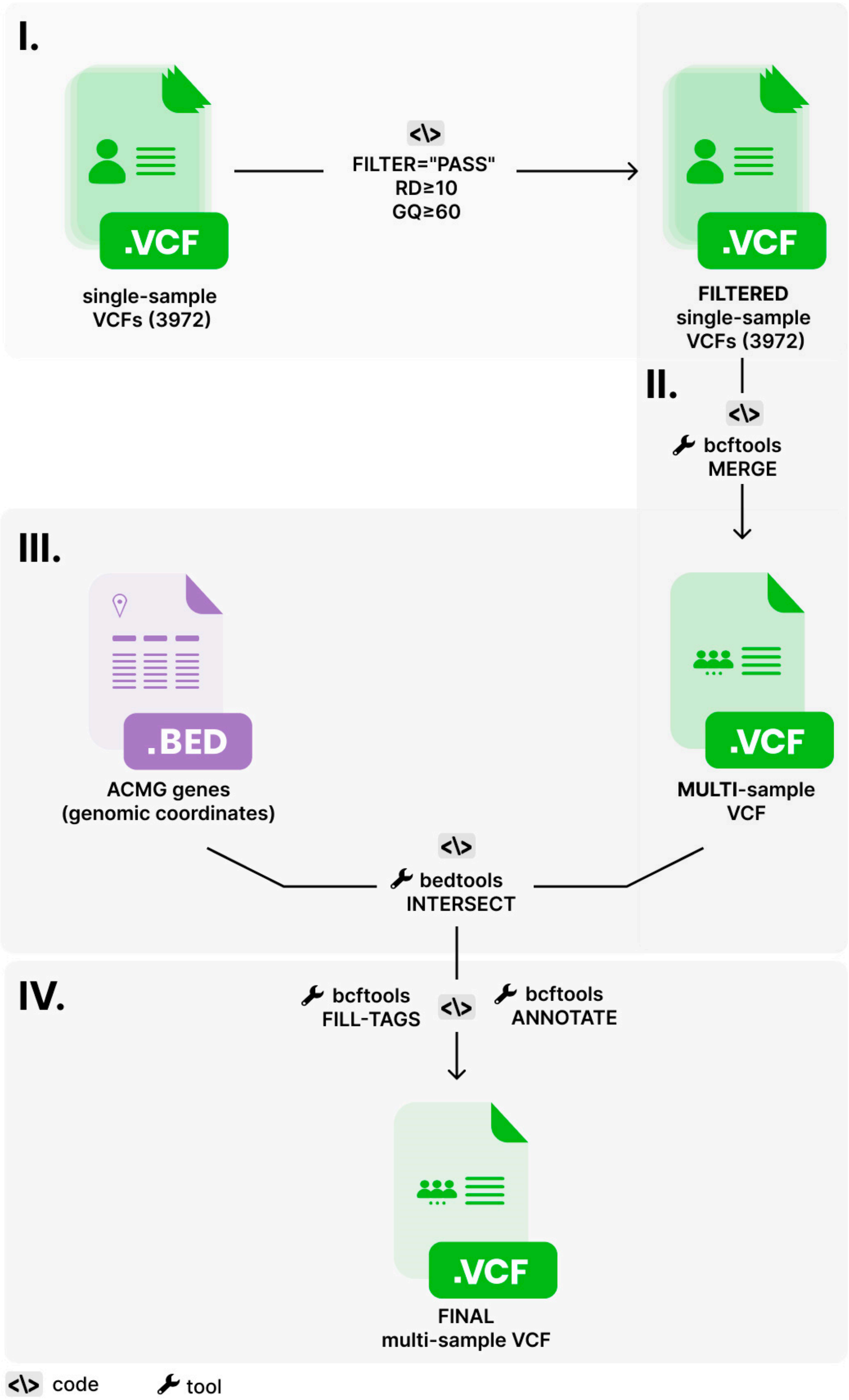

Figure S1. Pipeline used for processing and generating multi-sample VCF file. RD: read depth; GQ: genotype quality; VCF: Variant Call Format file.
