# Supplementary material for: Medically Actionable Secondary Findings from Whole-Exome Sequencing (WES) Data in a Sample of 3972 Individuals
Source: Int J Mol Sci. 2025 Apr 9;26(8):3509. doi: 10.3390/ijms26083509 (PMC12027037; doi:10.3390/ijms26083509)
Supplement: Supplementary file 1 [file ijms-26-03509-s001.zip › Supplementary data S1. References for Table S6A Gene specifications - variant interpretation details.pdf]

Supplementary References to support Table S6 gene specifications, variant interpretation specifications

CanVIG

S7.R1. https://www.cangene-canvaruk.org/_files/ugd/ed948a_f05b6f7767ad499a8539131d06035432.pdf

S7.R2. https://www.cangene-canvaruk.org/_files/ugd/ed948a_d6800c3321db40dd839644a9cd1634ff.pdf

S7.R3. https://www.cangene-canvaruk.org/_files/ugd/ed948a_10b5ca51dc4747af806065aefc69e3d7.pdf

S7.R4. https://www.cangene-canvaruk.org/_files/ugd/ed948a_41e68c523f434341996da021fc9c3009.pdf

S7.R5. https://www.cangene-canvaruk.org/_files/ugd/ed948a_828bce96646840f394e4fc5224210bd9.pdf

S7.R6. https://www.cangene-canvaruk.org/_files/ugd/ed948a_a5909620ea0d47d0a1bf81b97a8d82c0.pdf

ClinGen

S7.R7. https://cspec.genome.network/cspec/ui/svi/doc/GN077

S7.R8. https://cspec.genome.network/cspec/ui/svi/doc/GN009

S7.R9. https://cspec.genome.network/cspec/ui/svi/doc/GN003

S7.R10. https://cspec.genome.network/cspec/ui/svi/doc/GN022

S7.R11. https://cspec.genome.network/cspec/ui/svi/doc/GN013

S7.R12. https://cspec.genome.network/cspec/ui/svi/doc/GN002

S7.R13. Criteria Specification Registry (genome.network)

S7.R14 https://cspec.genome.network/cspec/ui/svi/doc/GN012

S4.R15. https://cspec.genome.network/cspec/ui/svi/doc/GN017
